# Mass and Force Sensing of an Adsorbate on a Beam Resonator Sensor

**DOI:** 10.3390/s150714871

**Published:** 2015-06-24

**Authors:** Yin Zhang, Ya-Pu Zhao

**Affiliations:** State Key Laboratory of Nonlinear Mechanics (LNM), Institute of Mechanics, Chinese Academy of Sciences, Beijing 100190, China; E-Mail: yzhao@imech.ac.cn

**Keywords:** mechanical resonator sensor, resonant frequency, inverse problem, beam

## Abstract

The mass sensing superiority of a micro-/nano-mechanical resonator sensor over conventional mass spectrometry has been, or at least is being firmly established. Because the sensing mechanism of a mechanical resonator sensor is the shifts of resonant frequencies, how to link the shifts of resonant frequencies with the material properties of an analyte formulates an inverse problem. Besides the analyte/adsorbate mass, many other factors, such as position and axial force, can also cause the shifts of resonant frequencies. The *in situ* measurement of the adsorbate position and axial force is extremely difficult if not impossible, especially when an adsorbate is as small as a molecule or an atom. Extra instruments are also required. In this study, an inverse problem of using three resonant frequencies to determine the mass, position and axial force is formulated and solved. The accuracy of the inverse problem solving method is demonstrated, and how the method can be used in the real application of a nanomechanical resonator is also discussed. Solving the inverse problem is helpful to the development and application of a mechanical resonator sensor for two reasons: reducing extra experimental equipment and achieving better mass sensing by considering more factors.

## 1. Introduction

Mass spectrometry is a widely-used analytical tool in biology and chemistry, which is also expected to play an important role in proteomics [1,2]. However, whether mass spectrometry can be the mainstay instrument in proteomics is questionable [2,3]. Although mass spectrometry has been used to identify protein species, the typical application involves the measurement of approximate $10^{8}$ molecules [3], which corresponds to several hundred kilodaltons (1 Dalton ≈ 1.65 × 10^−24^ g is approximately the mass of a proton or a neutron). To accelerate the identification of proteins, disease biomarkers and, thus, new drug development, the current demand is to characterize the proteome at the single-cell or single-molecule level [4,5], which is often beyond the mass range of conventional mass spectrometry [3]. In comparison, the nanoelectromechanical systems (NEMS)-based mechanical mass resonator has recently achieved the capability of detecting the mass of one Dalton [6]. Furthermore, mass spectrometry does not directly measure the mass of an analyte; it measures the mass-to-charge ratio (*m/z*) of the ionized analytes and the number of ions at each *m/z* value [1]. Therefore, during the application of mass spectrometry, there are three stages: ionization, separation and detection [7]. The structural change of a protein [3] or the damage of fragile biological macromolecules [8] caused by ionization is a serious problem in the application of mass spectrometry. Mass spectrometry also has the problem when being applied to small and thermostable compounds, because of the difficulty of ionization and transferring ionized analytes from the condensed phase into the gas phase [2]. On the other hand, the sensing mechanism of a mechanical mass resonator is the shifts of resonant frequencies, which can work with electrically-neutral analytes. The first two stages of ionization and separation are thus unnecessary for a mechanical mass resonator [4].

Moore’s law, which states that the number of components in integrated circuits doubles every year [9] and chip performance doubles every eighteen months, has successfully and succinctly predicted/summarized the revolutionary development of computer science and technology since the 1960s. In 2005, the researchers from the Oak Ridge National Laboratory asserted that the development of a micro-/nano-electromechanical systems (MEMS/NEMS)-based mechanical mass resonator/sensor “is poised for such a revolution” (as described by Moore’s law) [10]. The rapid development of a mechanical mass resonator sensor even causes Moore’s law underestimate its progress in performance: the mass sensing resolution has been steadily improving by approximately an order of magnitude per year [4]. The ultimate goal of any detection method is to achieve the level of resolving a single quantum of a measured entity [11]. This goal is also a major driving force for the development of a mechanical mass resonator sensor as reflected by the following observation: many approaches emphasize the minimum number of target species or labels that can be detected, the “single-molecule” detection is often the implicit goal [12]. An essential idea of improving the mass sensing resolution of a mechanical resonator sensor is to increase its resonant frequencies, and therefore, a small fractional change in large resonant frequency is still absolutely large enough to be detected [8]. Because the resonant frequency of a beam is proportional to $h/L^{2}\times \sqrt{E/\rho }$ (h and L are the thickness and length; E and ρ are Young’s modulus and mass density) [13,14,15], there are two major approaches to increase the resonant frequencies of a mechanical resonator sensor: The first is to reduce the structure dimensions, which makes the factor of $h/L^{2}$ larger, and at the same time, the fractional change in mass is also larger in a smaller resonator for the same analyte. The second is to use materials with large E/ρ, such as carbon nanotube (CNT) [6,16,17] and graphene [14]. The miniaturized mechanical resonator can vibrate with a resonant frequency of 2 GHz (1 GHz = $10^{9}$ Hz) [6] or even higher. For a mechanical resonator with a sub-micron length scale and GHz resonant frequency, measuring its motion and maintaining the frequency resolution at the level of parts-per-billion (ppb) are extremely challenging, because the thermal fluctuation effect stands out [18]. Although cryogenically cooling the nanomechanical resonator at liquid helium temperature is always effective for reducing/suppressing thermal noise, huge efforts are still needed to refine the read-out circuitry design [6,19]. Micro-/nano-mechanical mass resonators with the mass sensing resolution of detecting the presence of a cell [20], a virus [21], a protein [3,4], a molecule [6,22] and an atom [16,17] have been developed. Despite those marvelous achievements, the mechanical mass resonator actually has the problem of measuring the mass of an analyte, even though it has the capability of detecting the smaller resonant frequency shift induced by a molecule/atom [3,17].

The reason is that the analyte mass and its position on a resonator are the two convolving factors determining the shifts of resonant frequencies [3,8,17]. For any given shift of resonant frequency, there are infinite possible combinations of mass and position [14]. To know the mass, the position must be known (as for a forward problem). For an analyte as small as a molecule/atom, detecting the position is extremely difficult, which has been deemed as the most important problem for a nanomechanical resonator sensor [8]. In addition, the fact that adsorption not only adds mass, but also changes stiffness further complicates the problem [23]. The stiffness change mainly results from the following three mechanisms: (1) the adsorbate stiffness: when adsorbates form a layer with a finite thickness, the stiffness of the resonator-adsorbate layer composite structure is dependent on Young’s modulus and the thickness of the adsorbate layer [23,24]; (2) the change of mechanical properties: because chemical bonds or others can form or break during adsorption, mechanical properties, such as Young’s modulus and Poisson’s ratio, change correspondingly; for example, the formation of an amalgamation in the mercury adsorption test [25,26] and the formation of hydride in the hydrogen adsorption test [27,28]; the partial dissolution of the polymer coated on a silicon resonator owing to the adsorption and diffusion of analytes [29]; and (3) the change of stress, which finally leads to the change of the resonator axial force. When adsorbates stay on the resonator surface, the electrostatic [30] or Lennard–Jones [31] interactions of adsorbate–adsorbate and adsorbate–resonator surface atom result in surface stress, which generates both the effects of axial loading and bending moment [32]. The axial loading effect is responsible for the stiffness change [32]. The chemical reaction inside a microfluidic channel embedded in a silicon nitride-based resonator alters the surface stress at the solid-liquid interface [33]. Because the adsorbates and resonator material absorb light differently [34,35], thermal (axial) stress under laser irradiation is induced. When adsorbates diffuse into the resonator material, the interactions between adsorbates-resonator atoms can also cause the stress change. For example, after adsorption on a gold-palladium alloy surface, the hydrogen molecule dissociates into atomic hydrogen, which then diffuses into the lattice of the alloy and forms an interstitial phase; the formation and expansion of interstitial hydrogen in the alloy lattice are believed to relieve the built-in tensile stress and, thus, reduce the resonant frequencies dramatically [36]. Similarly, the adsorption of water ions on a silicon resonator is believed to hydroxylate the surface, which relaxes the surface stress and is responsible for the decrease of resonant frequency [37]. In general, the stiffness change should be taken into account together with the mass loading effect. Otherwise, the experimental data will be wrongly interpreted or even uninterpretable [23,26]. Two vivid examples are that in the acetylene adsorption test on a silicon resonator [38] and the pentacene adsorption test on a graphene resonator [39], the resonant frequencies of both resonators increase. Because mass loading decreases the resonant frequency, the increase of resonant frequency can only mean that the stiffness change is the dominant effect.

The resonator with an adsorbate is often studied as a forward/direct problem, in which the mass and position of an adsorbate are given to see how the resonant frequencies vary [40,41,42,43]. In the real application of a mass resonator, the resonant frequencies are the measured quantities; the mass and position of an adsorbate are unknown. Therefore, an inverse problem arises naturally: how to use the resonant frequencies to determine the mass and position? There are very few studies on this inverse problem. Hanay *et al.* [3] and Jensen *et al.* [17] solved the inverse problem by building the histograms of event probability *versus* frequency shift for the ensembles of sequential single protein/atom adsorption; the mass of the adsorbed protein/atom can be told with a certain confidence level. To accurately “decouple” the mass and position of an adsorbate, the statistics method requires tens or hundreds of adsorption events to build the histogram [3,17]. In comparison, a rather straightforward method was presented to solve the inverse problem of determining the mass and position of an adsorbate on a beam [44] and a string [45] by the shifts of resonant frequencies. The inverse problem is approximately solved by the Rayleigh–Ritz method by assuming that the beam/string strain energy does not change after mass loading/adsorption and is (approximately) equal to the kinetic energy of the unloaded beam/string [44,45]. Although it can be a good approximation in certain circumstance, the assumption in general is not valid, which could be the very reason why the method does not work when an adsorbate is (very) close to the cantilever clamped end or its mass is (very) small [44]. An improved method, which incorporates damping and is capable of handling the scenario when an adsorbate is close to the clamped end, was thus proposed [46]. Furthermore, as a result of the assumption, the beam bending stiffness [44] and the string tension [45] do not appear at all; their inverse problem method thus cannot be used to solve the stiffness change case. Tension is incorporated as an important parameter in the inverse problem of an adsorbate on a circular membrane, and the inverse problem is solved by assuming the adsorbate-induced tension is very small compared with the original one [14]. In the above inverse problem solving methods [3,14,17,44,45,46], the very key assumption is that an adsorbate only causes the mass-loading effect. In this study, the effect of stiffness change (due to axial load) is added; the mass, position and axial load are the three unknowns in a beam resonator. More importantly, a systematic method of formulating and solving the inverse problem is presented. Because solving the inverse problem can be very difficult and time consuming, most nanomechanical resonators cannot perform real-time mass sensing [3]. The general method presented in this study provides a straightforward and relatively fast way of solving the inverse problem, which should be of some help to mass sensing in real time.

## 2. Model Development

**Figure 1:**
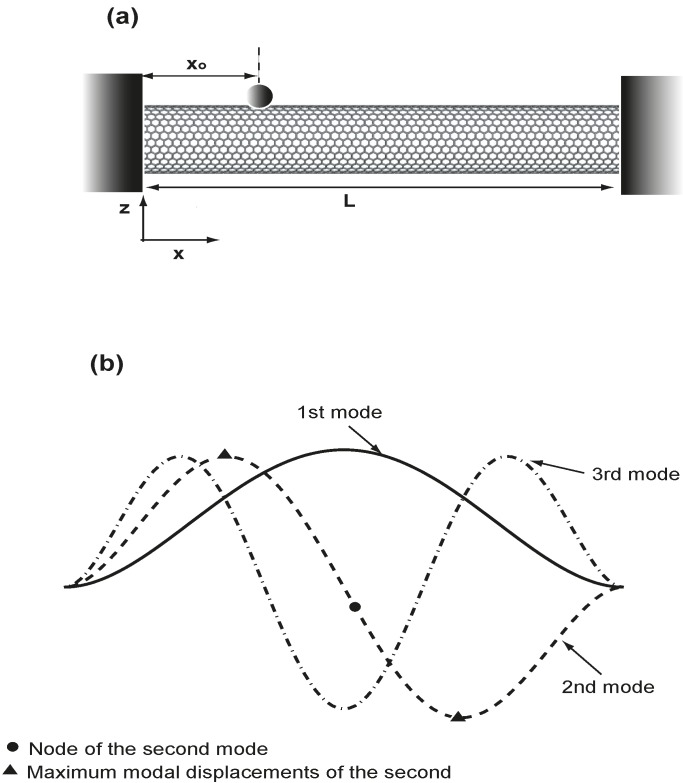
(**a**) Schematic diagram of an adsorbate on a carbon nanotube-based resonator; (**b**) the first three modes of a uniform clamped-clamped beam.

Figure 1a is the schematic of an adsorbate on a carbon nanotube (CNT)-based resonator with a length of L. The governing equation of the resonator, which is modeled as a beam, is given as follows [15,40,41]:

(1) $$[m+M_{o}\delta (x-x_{o})]\frac{\partial^{2}w}{\partial t^{2}}-T\frac{\partial^{2}w}{\partial x^{2}}+D\frac{\partial^{4}w}{\partial x^{4}}=0$$

where m is the resonator mass per unit length; $M_{o}$ and $x_{o}$ are the mass and position of the adsorbate, which is modeled as a concentrated mass by the Dirac delta function of δ; [15,40,41]. w is the beam displacement; T is the axial load; T > 0 is tension; and T < 0 is compression. T can vary due to adsorption. D is the beam bending stiffness, and D=EI (E and I are the beam’s Young’s modulus and the moment of inertia, respectively).

By introducing ξ=x/L, $\tau =\sqrt{EI/(mL^{4})}t$ and W=w/L [15,40], Equation (1) is non-dimensionalized as follows:

(2) $$[1+\alpha \delta (\xi -\xi_{o})]\frac{\partial^{2}W}{\partial \tau^{2}}-\beta \frac{\partial^{2}W}{\partial \xi^{2}}+\frac{\partial^{4}W}{\partial \xi^{4}}=0$$

where the dimensionless parameter $\alpha =M_{o}/(mL)$ is the ratio of the adsorbate mass to that of the resonator; $\beta =TL^{2}/D$ is the ratio of the axial load to the beam transverse stiffness; $\xi_{o}=x_{o}/L$ is the adsorbate location. When the compressive axial load reaches a critical value, the beam buckles. Equation (2) is a linear equation, which cannot describe the beam vibration in the post-buckling region. For Equation (2) to apply, $\beta >-4\pi^{2}$ for the clamped-clamped beam and $\beta >-\pi^{2}/4$ for the cantilever beam [47] are required.

The Galerkin method is an efficient method for the eigenfrequency computation of a beam with small concentrated masses [40], which assumes the following form for W(ξ,τ):

(3) $$W(\xi ,\tau )=\sum_{j=1}^{N}a_{j}(\tau )\phi_{j}(\xi )$$

where N is the mode number and $a_{j}(\tau )$ is the unknown j-th modal amplitude. $\phi_{j}(\xi )$ is the j-th mode of a uniform clamped-clamped beam [47]; the first three mode shapes of the clamped-clamped beam are presented in Figure 1b. Substitute Equation (3) into Equation (2), time $\phi_{i}(\xi )$ and integrate from 0 to 1; the following governing equations are derived:

(4) $$M\ddot{q}+Kq=0$$

Here, $\overset{•}{\left(\right)}=\partial /\partial \tau$, and q is a vector given as $q={\left(a_{1},a_{2},\cdots \cdots ,a_{N}\right)}^{T}$. M and K are the N×N matrices of mass and stiffness, respectively, which are given as the following by using both the orthonormality property of $\phi_{j}(\xi )$ and the integration property of the Dirac function [15,40]:

(5) $$M_{ij}=\delta_{ij}+\alpha \phi_{i}(\xi_{o})\phi_{j}(\xi_{o}),\;K_{ij}=\kappa_{j}^{4}\delta_{ij}-\beta \int_{0}^{1}\phi_{i}(\xi )\frac{\partial^{2}\phi_{j}(\xi )}{\partial \xi^{2}}d\xi$$

where $\delta_{ij}$ is the Kronecker delta function, and it is noticed that the presence of the concentrated mass (α) makes the mass matrix non-diagonal; $\kappa_{j}^{2}$ is the *j*-th (dimensionless) eigenfrequency of a uniform undamped beam with no axial load. Clearly, the presence of the axial load (β) has a direct impact on the stiffness matrix K, which also leads to the variation of the resonant frequencies. The first three $\kappa_{j}^{2}$ of a clamped-clamped beam are given as follows [48]:

(6) $$\omega_{1}^{o}=\kappa_{1}^{2}=4.73^{2}=22.3733,\;\omega_{2}^{o}=\kappa_{2}^{2}=7.8532^{2}=61.6728,\;\omega_{3}^{o}=\kappa_{3}^{2}=10.9956^{2}=120.9034$$

To find out the resonant frequencies of the beam with the concentrated mass and axial load, $a_{j}(\tau )=b_{j}e^{i\omega \tau }$ ($b_{j}$ is the unknown constant and ω is the resonant frequency) is assumed and substituted into Equation (4), which leads to the following α eigenvalue problem:

(7) $$K-M\omega^{2}=0$$

To find the eigenfrequency/resonant frequency of ω, the adsorbate mass (α), location (ξ_o_) and axial load (β) are needed. Here, up to three resonant frequencies are calculated; N≥3 is required. By carefully choosing the lower and upper bounds for each resonant frequency, different ω are solved one by one by the Newton–Raphson method [40,49].

## 3. Results and Discussion

**Figure 2:**
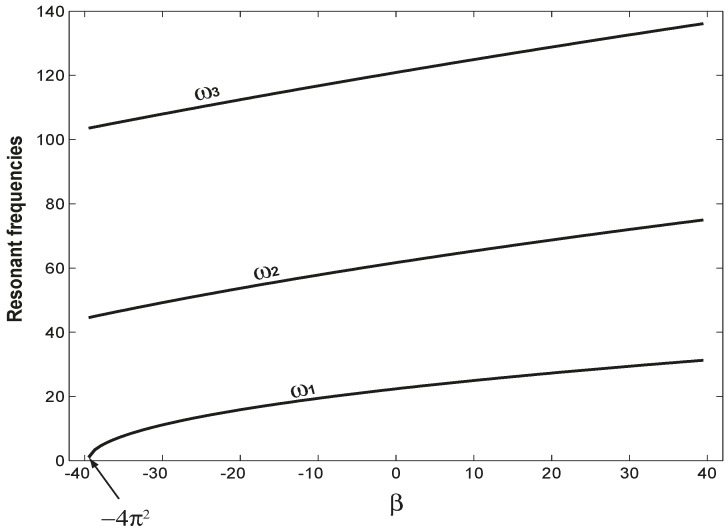
The variations of the first three resonant frequencies as the axial load (β) varies and α=0. At $\beta =-4\pi^{2}$, the first resonant frequency ($\omega_{1}$) becomes zero, which indicates buckling.

Many mechanical resonators are the clamped-clamped (C-C) beam structure [3,4,6,19], which has the highest resonant frequencies among all beam structures. Here, the C-C beam is studied. Figure 2 examines how the first three resonant frequencies vary as the axial load (β) changes. In Figure 2, there is no adsorbate, *i.e.*, α = 0 is set. Clearly, all of the resonant frequencies increase monotonically as β increases. At $\beta =-4\pi^{2}$, the first resonant frequency ($\omega_{1}$) is zero, which indicates buckling. If a closer look is taken, we can find that the different resonant frequencies vary differently as the axial load changes. For example, when β = 0, the three resonant frequencies are given by Equation (6) as $\omega_{1}^{o}$ = 22.3733, $\omega_{2}^{o}$ = 61.6728 and $\omega_{3}^{o}$ = 120.9034; at β = 10, the three resonant frequencies are $\omega_{1}$ = 24.9591, $\omega_{2}$ = 65.2996 and $\omega_{3}$ = 124.9291. The corresponding frequency change (defined as $\Delta \omega_{i}=\omega_{i}-\omega_{i}^{o}$) and its percentage (defined as $\Delta \omega_{i}/\omega_{i}^{o}$) are: $\Delta \omega_{1}$ = 2.5858 (11.56%), $\Delta \omega_{2}$ = 3.6268 (5.88%) and $\Delta \omega_{3}$ = 4.0257 (3.33%). That different resonant frequency has different sensitivity to the axial load has been noticed [50] and used as a mechanism to detect the surface stress inside a micro-/nano-structure [13].

**Figure 3:**
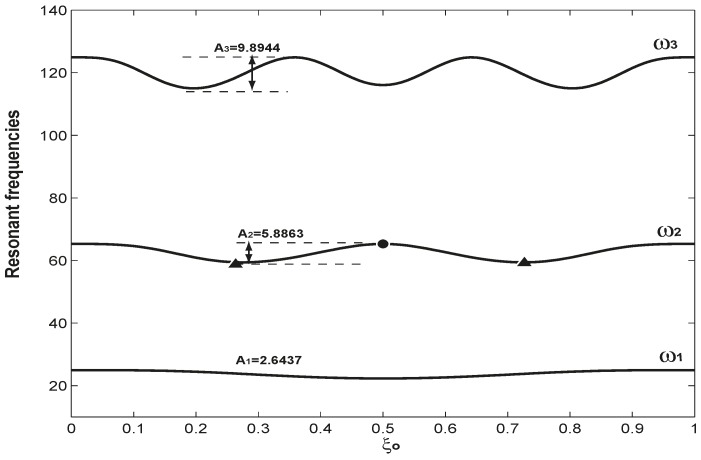
The variations of the first three resonant frequencies as an adsorbate moves from one clamped end to the other. Here, the mass and axial load are fixed as α=0.1 and β=10. $A_{1}$, $A_{2}$ and $A_{3}$ are the amplitudes of the three resonant frequencies, which indicate the difference between the maximum and minimum of those frequencies.

Figure 3 examines the variations of the first three resonant frequencies as the adsorbate moves from one end to the other. In Figure 3, α = 0.1 and β = 10 are fixed; $\xi_{o}$ varies from zero to one. Again, the three resonant frequencies respond differently as the adsorbate moves from one end to the other. As seen in Equation (5), the adsorbate actual mass (α) and its location ($\xi_{o}$) are the two intricate factors determining the effective mass for the system. The variation patterns of the three resonant frequencies are actually based on the mode shapes, as presented in Figure 1b. At the boundaries of ξ = 0, 1 and node(s) (*i.e.*, $\phi_{j}(\xi )$ = 0), the effective mass is zero, and the resonant frequencies are thus the maximum. For the first mode, there is no node, and its modal displacement reaches the maximum at $\xi_{o}$ = 0.5, which corresponds to the maximum effective mass and, thus, minimum resonant frequency. For the second mode, which has one node at ξ = 0.5 and is marked as a solid circle in both Figure 1b and Figure 3, the resonant frequency reaches its maximum because $\phi_{2}(0.5)$ = 0, and the effective mass is zero. At the same time, the modal displacement of the second mode reaches the maximum at ξ = 0.27 and ξ = 0.73, which are marked as two solid triangles in Figure 1b and Figure 3; the effective mass becomes maximum, and the second resonant frequency thus reaches its minimum. The node at ξ = 0.5 and the two maximum modal displacements at ξ = 0.27 and ξ = 0.73 are responsible for the variation of the second resonant frequency, as presented in Figure 3. A similar analysis can be applied to explain the variation of the third resonant frequency. Here, $A_{i}$ is defined as the difference of the maximum and minimum of the i-th resonant frequency, and $A_{1}$ = 2.6437, $A_{2}$ = 5.8863 and $A_{3}$ = 9.8944. The fact that $A_{3}$ > $A_{2}$ > $A_{1}$ indicates that a higher mode has higher mass sensitivity, which has been used as a mechanism to detect the mass and location of an accreted particle on a micromechanical resonator [51]. In summary, Figure 2 and Figure 3 demonstrate two things: (1) that the axial load and mass have different impacts on different resonant frequencies; (2) that for given axial load and mass (including its position), different resonant frequencies respond differently. These two things are the very physical mechanism to solve the inverse problem.

Now, let us present how to use the mechanism to solve the inverse problem. Here, the computation example of α = 0.1, $\xi_{o}$ = 0.3 and β = 10 is given, which results in the following three resonant frequencies, as given by Equation (7):

(8) ω_1_ = 23.5217, ω_2_ = 59.5752, ω_3_ = 121.8384

As shown in Figure 2, tension stiffens the beam and, thus, increases the resonant frequencies; on the other hand, the adsorbate mass always reduces the resonant frequencies. Compared with the three resonant frequencies of α = β = 0 as given in Equation (6), the competition between tension and mass leads to the decrease of the second resonant frequency and the increase of the first and third ones. In both Figure 2 and Figure 3, the eigenfrequencies are solved as a forward problem by supplying α, $\xi_{o}$ and β to Equation (7). However, in the real application of the resonator sensor, the resonant frequencies are the measured quantities, which in this computation example, are given in Equation (8); α, $\xi_{o}$ and β in general are the unknowns to be determined. In order to present a better and graphic illustration of how the inverse problem is solved, we start with the simpler case of two variables. In this case, β = 10 is known, and α and $\xi_{o}$ are the two unknowns to be determined. Because the original axial load (or surface stress) can be determined during an experimental calibration process by measuring the shift of a resonant frequency [52], this inverse problem solving technique for two variables can correspondingly be applied to the case that adsorption induces no surface stress.

**Figure 4:**
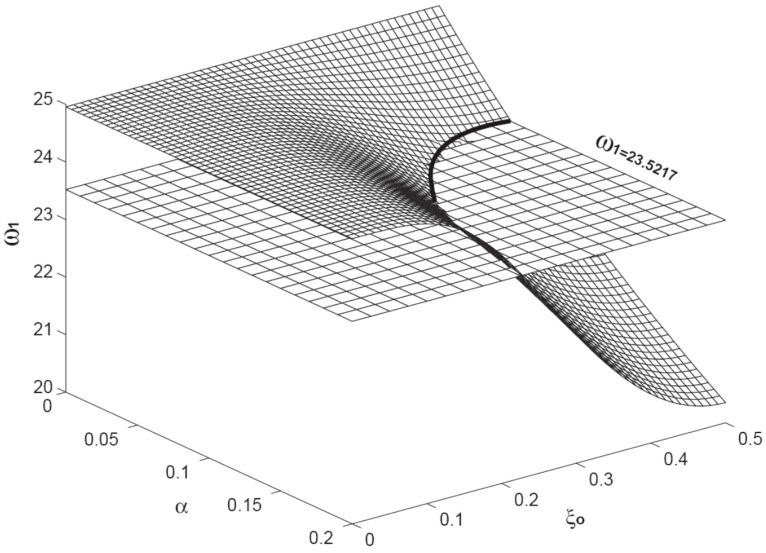
The variation of the first resonant frequency ($\omega_{1}$) as a function of α and $\xi_{o}$. The level plane is the one with the constant of $\omega_{1}$ = 23.5217. The intersection of the two planes is marked with a solid curve. Here, the axial load is fixed as β = 10.

**Figure 5:**
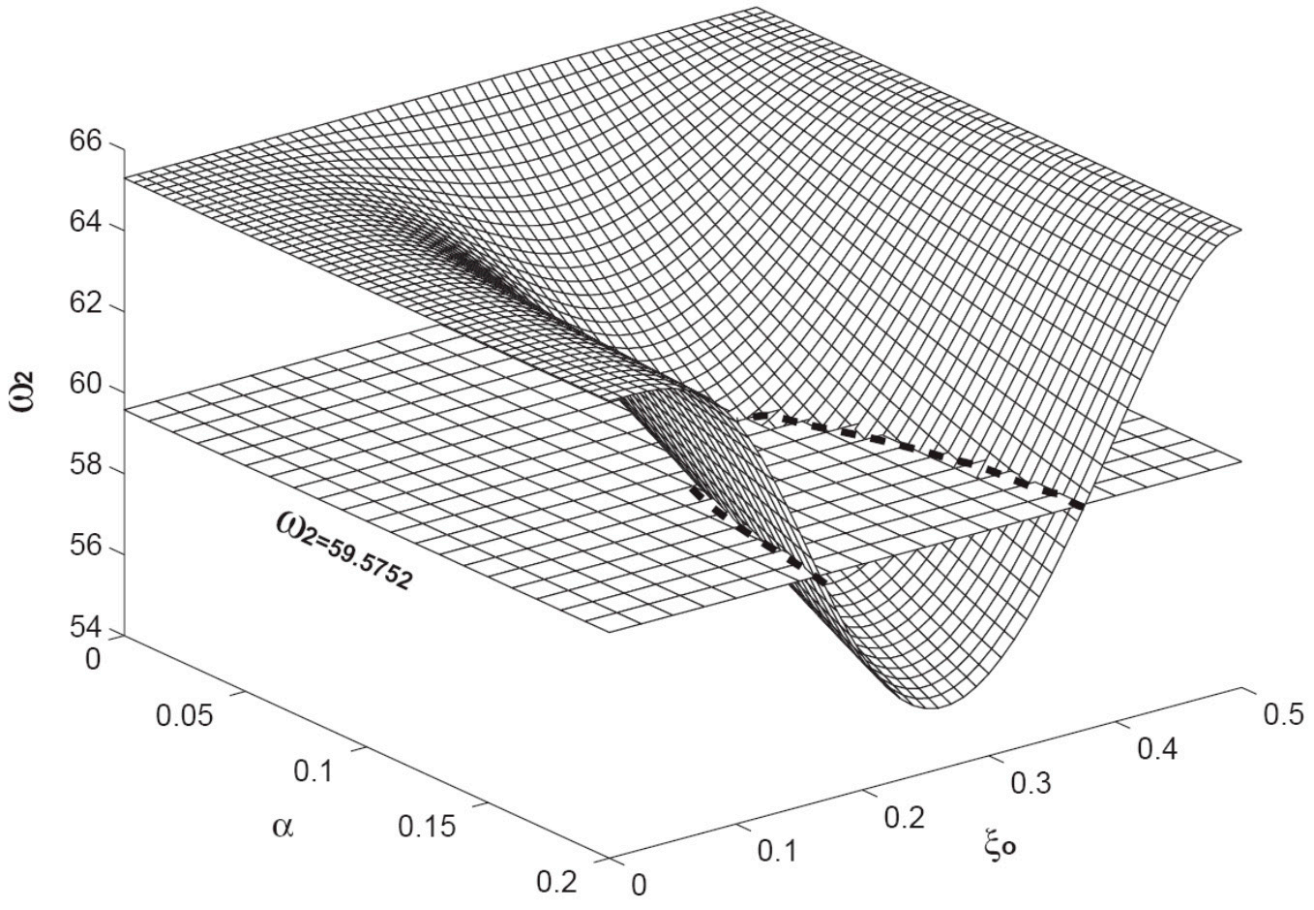
The variation of the second resonant frequency ($\omega_{2}$) as a function of α and $\xi_{o}$. The level plane is the one with the constant of $\omega_{2}$ = 59.5752. The intersection of the two planes is marked with a dashed curve. Here, the axial load is fixed as β = 10.

**Figure 6:**
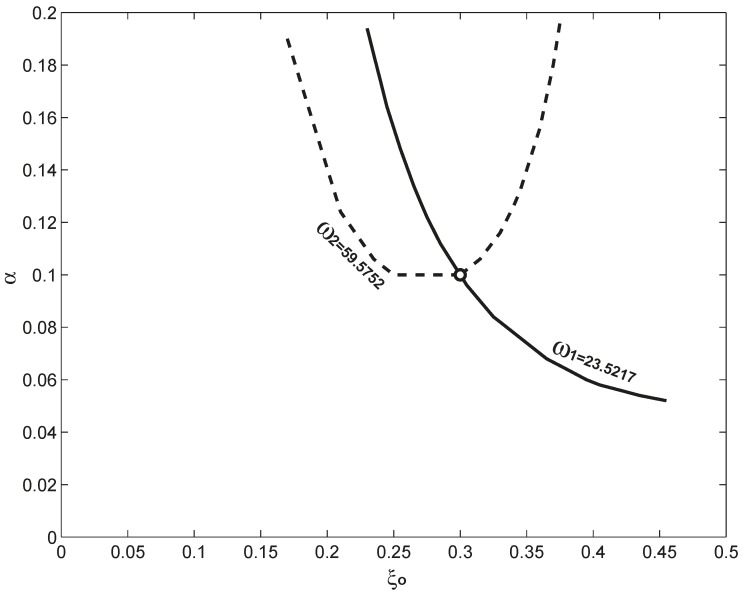
The projections of the two intersection curves obtained in Figure 4 and Figure 5 into the α − $\xi_{o}$ plane. The intersection of the two curves is marked with a circle, which corresponds to (α, $\xi_{o}$) = (0.1, 0.3) exactly.

Figure 4 presents the variation of the first resonant frequency ($\omega_{1}$) as the function of α and $\xi_{o}$. Here, α varies from zero to 0.2; $\xi_{o}$ varies from zero to 0.5. Because the C-C beam is a symmetric structure, the adsorbate at $\xi_{o}$ and 1 − $\xi_{o}$ results in the same change for any arbitrary resonant frequency. Therefore, only half of the beam span is examined here. The level plane is the one with $\omega_{1}$ = 23.5217. The intersection of the two planes are marked with a solid line, which indicates the combinations of α and $\xi_{o}$ resulting in the same first resonant frequency of $\omega_{1}$ = 23.5217. This solid line also indicates that the combinations are infinite. Figure 5 presents the variation of the second resonant frequency ($\omega_{2}$) as the function of α and $\xi_{o}$. The level plane is the one with $\omega_{2}$ = 59.5752. Again, the intersection of the two planes is the combination of α and $\xi_{o}$ resulting the same second resonant frequency of $\omega_{2}$ = 59.5752, which is marked as a dashed line. Once again, the dashed line indicates that the infinite combinations of α and $\xi_{o}$ result the same second resonant frequency of $\omega_{2}$ = 59.5752. When α, $\xi_{o}$ and β are given, each eigenfrequency is uniquely determined by Equation (7) as a forward problem. In comparison, in this two-variable case of the inverse problem, for a given eigenfrequency, there are infinite combinations of α and $\xi_{o}$. However, when these two curves obtained in Figure 4 and Figure 5 are projected into the $\xi_{o}$− α plane, they intersect, and in Figure 6, the intersection point is marked as a circle, which is exactly (α, $\xi_{o}$) = (0.1, 0.3). Physically, the reason for the two curves to intersect is that the mechanism mentioned above: α and $\xi_{o}$ have different impacts on different resonant frequencies; different resonant frequencies respond differently to the given α and $\xi_{o}$. Mathematically, as seen in Equation (5), α is a coefficient, and $\xi_{o}$ is embedded in the function of the mode shape in the mass matrix.

The above graphic solution is carried out by using Equation (7) to compute two eigenfrequencies in the possible regions of 0 ≤ α ≤ 0.2 and 0 ≤ $\xi_{o}$ ≤ 0.5. There is a more succinct way of summarizing this solution process. Equation (7) actually has the function form of $F(\omega ,\alpha ,\xi_{o},\beta )=K(\beta )-M(\alpha ,\xi_{o})\omega^{2}=0$, which is a transcendental equation. In the forward problem, α, $\xi_{o}$ and β are given; ω are then computed one by one. In the above inverse problem solving process, two resonant frequencies ($\omega_{1}$, $\omega_{2}$) and an axial load of β = 10 are known, which in essence gives the following two equations:

(9) $$\left\{ \begin{array}{l} F(\omega_{1},\alpha ,\xi_{o},\beta )=F(23.5217,\alpha ,\xi_{o},10)=0\\ F(\omega_{2},\alpha ,\xi_{o},\beta )=F(59.5752,\alpha ,\xi_{o},10)=0 \end{array}\right.$$

Here, Equation (9) offers two (nonlinear) equations to solve the two unknowns of α and $\xi_{o}$. Again, the Newton–Raphson method [49] is used, and the exact solution of (α, $\xi_{o}$) = (0.1, 0.3) is also obtained. To solve the two unknowns, their two initial values need to be guessed [49], and here, the Newton–Raphson method is not sensitive to the initially guessed values. The first two resonant frequencies ($\omega_{1}$, $\omega_{2}$) are used in Equation (9). An alternative is to use the first and third resonant frequencies ($\omega_{1}$, $\omega_{3}$), which gives the following two equations:

(10) $$\left\{ \begin{array}{l} F(\omega_{1},\alpha ,\xi_{o},\beta )=F(23.5217,\alpha ,\xi_{o},10)=0\\ F(\omega_{3},\alpha ,\xi_{o},\beta )=F(121.8384,\alpha ,\xi_{o},10)=0 \end{array}\right.$$

Equation (10) also obtains the exact solution of α and $\xi_{o}$. The second and third resonant frequencies ($\omega_{2}$, $\omega_{3}$) can also be used as another alternative.

As for the case in which adsorption induces axial load, there are three unknowns of α, $\xi_{o}$ and β. Similarly, the following three equations can be given to solve the three unknown:

(11) $$\left\{ \begin{array}{l} F(\omega_{1},\alpha ,\xi_{o},\beta )=F(23.5217,\alpha ,\xi_{o},10)=0\\ F(\omega_{2},\alpha ,\xi_{o},\beta )=F(59.5752,\alpha ,\xi_{o},10)=0\\ F(\omega_{3},\alpha ,\xi_{o},\beta )=F(121.8384,\alpha ,\xi_{o},10)=0 \end{array}\right.$$

The Newton–Raphson method now shows the sensitivity to the initial guess. Here, our initial guess is (α, $\xi_{o}$, β) = (0.069, 0.23, 5.1), which leads to the (almost) exact solution of (α, $\xi_{o}$, β) = (0.1, 0.3, 10). However, if the initial guess significantly deviates from the real one, the Newton–Raphson method cannot find the solution. It is possible that for the given three resonant frequencies of Equation (8) there are other solutions than (α, $\xi_{o}$, β) = (0.1, 0.3, 10). However, it is extremely difficult to use the Newton–Raphson method to scout out and find all of the possible solutions, because it is hard to get a proper initial guess. The method used in Figure 4 and Figure 5 can help to find the solution with a high computational price to pay. In Figure 4 and Figure 5, α increases from zero to 0.2 by 50 steps, and $\xi_{o}$ increases from zero to 0.5 by 50 steps. To obtain the two resonant frequencies, the total 2 × 50 × 50 = 5000 times eigenvalue computations of Equation (7) were conducted. For three variables, if β increases from zero to 20 by 50 steps, the total 3 × 50 × 50 × 50 = 3.75 × 105 times eigenvalue computations are now required. In the range (of 0 ≤ α ≤ 0.2, 0 ≤ $\xi_{o}$ ≤ 0.5 and 0 ≤ β ≤ 20), no solution other than (α, $\xi_{o}$, β) = (0.1, 0.3, 10) can be found. More hunting for the solution can be done by enlarging the ranges of α and β, which also requires many more computations. However, it should not be surprising that such difficulty is encountered in the three-variable case. An analogy is that even the complex approaches based on pattern recognition algorithms and sensor arrays failed to detect a mixture of three unknown chemical vapors [34]. Mathematically speaking, the essential difficulty results from that the nonlinear equation set of Equation (11) can have more than one solution set of α, $\xi_{o}$ and β for three given resonant frequencies, especially when the adsorbate position ($\xi_{o}$) is close to a clamped end. Physically, when an adsorbate is close to an end, its mass effect become small; as seen in Figure 3, there is only a tiny change of resonant frequency, which also causes the computation accuracy issue. Schmid *et al.* [45] also observed the similar problem that the model error becomes larger when an adsorbate is closer to a string end, and their computation excluded all cases of $\xi_{o}$ < 0.2 to ensure accuracy. The Newton–Raphson method seems insensitive to the initial guess of and β. Besides the adsorbate position, the experimental measurement error is also a major source determining the accuracy of the above inverse problem solving method.

In Equation (11), the three measured resonant frequencies of $\omega_{1}$, $\omega_{2}$ and $\omega_{3}$ are input as the exact values.

In a real experiment, the measurement error is unavoidable. To study the impact of the experimental measurement error on the results, the following three resonant frequencies are given (arbitrarily):

(12) ω_1_ = 24.4626, ω_2_ = 61.3624, ω_3_ = 124.2752

Compared with the exact ones of Equation (8), the errors of $\omega_{1}$, $\omega_{2}$ and $\omega_{3}$ are 4%, 3% and 2%, respectively. Now, these three erroneous resonant frequencies are used as the input, and Equation (11) changes correspondingly as follows:

(13) $$\left\{ \begin{array}{l} F(\omega_{1},\alpha ,\xi_{o},\beta )=F(\text{24}.\text{4626},\alpha ,\xi_{o},10)=0\\ F(\omega_{2},\alpha ,\xi_{o},\beta )=F(\text{61}.\text{3624},\alpha ,\xi_{o},10)=0\\ F(\omega_{3},\alpha ,\xi_{o},\beta )=F(\text{124}.\text{2752},\alpha ,\xi_{o},10)=0 \end{array}\right.$$

To solve the above equation, the same initial guess of (α, $\xi_{o}$, β) = (0.069, 0.23, 5.1) is used, and Equation (13) yields the result of (α, $\xi_{o}$, β) = (0.09161, 0.30819, 14.03073). In comparison with the exact values of (α, $\xi_{o}$, β) = (0.1, 0.3, 10), the corresponding errors of α, $\xi_{o}$ and β are −8.39%, 2.73% and 40.31%, respectively. Clearly, the tension (β) error is significantly larger compared with the other two. Because the three resonant frequencies as given in Equation (12) are consistently higher, a larger β is a natural compensating mechanism. Physically, because β = 10 is a rather small tension whose magnitude is about one fourth of the buckling load, as seen in Figure 2, it has to increase dramatically for the higher input “measured” resonant frequencies. Mathematically, the relation of resonant frequencies with these three parameters is highly nonlinear, which is responsible for the different errors and sensitivities to the measurement errors. To further demonstrate this sensitivity issue, the following example is presented, which gives different “measured” resonant frequencies

(14) ω_1_ = 24.2273, ω_2_ = 60.7667, ω_3_ = 123.0568

Now, the errors of $\omega_{1}$, $\omega_{2}$ and $\omega_{3}$ are 3%, 2% and 1%, respectively. Compared with those of Equation (12), each decreases by 1%. Again, by substituting these “newly measured” resonant frequencies into Equation (11), the following equation is obtained:

(15) $$\left\{ \begin{array}{l} F(\omega_{1},\alpha ,\xi_{o},\beta )=F(\text{24}.\text{2273},\alpha ,\xi_{o},10)=0\\ F(\omega_{2},\alpha ,\xi_{o},\beta )=F(\text{6}0.\text{7667},\alpha ,\xi_{o},10)=0\\ F(\omega_{3},\alpha ,\xi_{o},\beta )=F(\text{123}.0\text{568},\alpha ,\xi_{o},10)=0 \end{array}\right.$$

Now, by using the same initial guess, Equation (15) gives (α, $\xi_{o}$, β) = (0.09283, 0.30015, 12.69567), and the corresponding errors are −7.17%, 0.05% and 26.96%, respectively. The errors of these three parameters all decrease, and the significant error reduction of β is also noticed. In many mass resonator sensor applications [3,4,6,16,17,39], mass (α) is actually the only target quantity to be measured. Therefore, it is also important to mention that the error of α is much smaller and less sensitive to the errors of the measured resonant frequencies as compared with that of β.

Because the model is for the one adsorbate case, we briefly discuss how this method can be used in a real application scenario. Firstly, the nanomechanical resonator can be cleaned by simply passing a large electrical current, which generates ohmic heating and boils off adsorbates [6,39]. Secondly, the nanomechanical resonator has achieved the sensitivity of detecting the shift of resonant frequency induced by the adsorption of a protein [3,4], a molecule [6] and an atom [16,17]. The step-wise resonant frequency variation physically indicates the discrete nature of the adsorbates arriving on the surfaces of a nanomechanical resonator one by one [3,4,6,16,17], which is also the hallmark of sensing an individual adsorption event [3].

## 4. Conclusions

That the inverse problem can be solved is based on the following two facts: (1) mass, position and axial load have different impacts on a given resonant frequency; (2) for a given mass, position and axial load, different resonant frequencies vary differently. By incorporating axial load, better mass sensing based on the model presented in this study is expected. The equation set as presented in Equation (11) gives a general formulation of the inverse problem. The first three lowest resonant frequencies are used to solve the inverse in this study. Because a higher mode has higher mass sensitivity, Equation (11) can be easily reformulated by simply supplying three other resonant frequencies. Although it has some numerical difficulties in some cases, the Newton–Raphson method offers a relatively fast solution to the inverse problem, which should be helpful to the real-time sensing application. The graphic solution procedure for the two-variable case is presented, and it can provide valuable information to guess the initial values for the Newton–Raphson method in the multiple-variable case. The trade-off is that much more computation is required.
